# Application of Recombinant Lactic Acid Bacteria (LAB) Live Vector Oral Vaccine in the Prevention of F4+ Enterotoxigenic *Escherichia coli*

**DOI:** 10.3390/vaccines12030304

**Published:** 2024-03-14

**Authors:** Jiangxu Yu, Jiyang Fu, Hongshuo Liu, Chao Kang, Zesong Wang, Yancheng Jin, Shuxuan Wu, Tianzhi Li, Ruicheng Yang, Meilin Jin, Huanchun Chen, Xiangru Wang

**Affiliations:** 1 National Key Laboratory of Agricultural Microbiology, College of Veterinary Medicine, Huazhong Agricultural University, Wuhan 430070, China; yujiangxu@webmail.hzau.edu.cn (J.Y.); a992534658@webmail.hzau.edu.cn (H.L.); wzs66@webmail.hzau.edu.cn (Z.W.); jinyancheng@webmail.hzau.edu.cn (Y.J.); wushuxuan@webmail.hzau.edu.cn (S.W.); litianzhi@webmail.hzau.edu.cn (T.L.); yangruicheng@mail.hzau.edu.cn (R.Y.); jinmeilin@mail.hzau.edu.cn (M.J.); chenhch@mail.hzau.edu.cn (H.C.); 2 Key Laboratory of Preventive Veterinary Medicine in Hubei Province, The Cooperative Innovation Center for Sustainable Pig Production, Wuhan 430070, China; 3 Wuhan Keqian Biology Co., Ltd., Wuhan 430070, China; fujy@webmail.hzau.edu.cn (J.F.); kangchao@kqbio.com (C.K.)

**Keywords:** enterotoxigenic *Escherichia coli* (ETEC), lactic acid bacteria (LAB), oral vaccine

## Abstract

Enterotoxigenic *Escherichia coli* (ETEC) causes severe diarrhea in piglets. The current primary approach for ETEC prevention and control relies on antibiotics, as few effective vaccines are available. Consequently, an urgent clinical demand exists for developing an effective vaccine to combat this disease. Here, we utilized food-grade *Lactococcus lactis* NZ3900 and expression plasmid pNZ8149 as live vectors, together with the secreted expression peptide Usp45 and the cell wall non-covalent linking motif LysM, to effectively present the mutant LTA subunit, the LTB subunit of heat-labile enterotoxin, and the FaeG of F4 pilus on the surface of recombinant lactic acid bacteria (LAB). Combining three recombinant LAB as a live vector oral vaccine, we assessed its efficacy in preventing F4+ ETEC infection. The results demonstrate that oral immunization conferred effective protection against F4+ ETEC infection in mice and piglets lacking maternal antibodies during weaning. Sow immunization during late pregnancy generated significantly elevated antibodies in colostrum, which protected piglets against F4+ ETEC infection during lactation. Moreover, booster immunization on piglets during lactation significantly enhanced their resistance to F4+ ETEC infection during the weaning stage. This study highlights the efficacy of an oral LAB vaccine in preventing F4+ ETEC infection in piglets by combining the sow immunization and booster immunization of piglets, providing a promising vaccination strategy for future prevention and control of ETEC-induced diarrhea in piglets.

## 1. Introduction

Enterotoxigenic *Escherichia coli* (ETEC) is a pathogenic strain of *Escherichia coli* that can cause diarrhea in humans and various animals. ETEC primarily affects young animals, spreading rapidly and resulting in severe diarrhea and dehydration in piglets under 7 days of age. It also affects piglets between 10 and 30 days of age, leading to stunted growth and gradual weight loss. Furthermore, ETEC is one of the main bacterial pathogens responsible for postweaning diarrhea (PWD) [1,2,3]. This pathogen causes significant economic losses to farms by inducing severe diarrhea and mortality in piglets. It is imperative to conduct research aimed at developing vaccines against this disease.

ETEC primarily colonizes the surface of the small intestinal mucosa and typically does not invade intracellularly. It relies on adhesins to bind specifically to receptors on intestinal epithelial cells [4], leading to diarrhea in animals through the secretion of enterotoxins, including heat-labile enterotoxin (LT) and heat-stable enterotoxin (ST). Therefore, adhesins and enterotoxins play a crucial role in the process of bacterial infection [5,6]. Adhesin primarily refers to the pili found on the surface of ETEC, which are filamentous polyprotein structures. Among the various types of ETEC pili, F4 (K88) pili have been extensively studied and are commonly observed in ETEC derived from pigs [7,8]. The key protein subunit responsible for forming the K88 pilus and serving as its adhesion component is FaeG [9]. Deletion of the FaeG subunit weakens the pathogenicity of F4+ ETEC, confirming its crucial role in ETEC infection and pathogenesis [10,11,12]. The complete LT toxin consists of one A subunit and five B subunits, with the A subunit being the primary virulence factor that induces severe diarrhea in animals [1,13,14]. Studies have demonstrated that mutating serine at position 63 to lysine and alanine at position 72 to arginine in the LTA subunit of heat-labile enterotoxin can reduce its toxicity [15,16].

Live vector vaccines employ molecular biology techniques to introduce the gene encoding the target antigen into a nonvirulent bacterial or viral vector. This process generates a recombinant strain capable of transmitting and replicating the target gene within the host organism, resulting in significant expression of the desired gene and subsequent induction of immune protective responses. *Lactococcus lactis*, a well-established strain of lactic acid bacteria (LAB), offers distinct advantages in terms of its rapid growth rate, operational simplicity, safety profile, and nontoxic nature. Moreover, the complete sequencing of its genome renders it an ideal candidate for expressing exogenous proteins and serving as a carrier for live vector vaccines [17,18,19]. However, LAB exhibit a significant degree of biological and genetic diversity, leading to variable expression levels of exogenous genes, including cases where there is no expression at all. Moreover, different host bacteria can display varying expression levels of the same genes, resulting in differences in cellular and humoral immune responses as well as variations in antigen presentation on recombinant strains’ surfaces. The level of protein expression plays a crucial role in determining the effectiveness of vector vaccines and subsequently affects immunized animals differently during practical use. This variability poses a challenge for the development of lactic acid bacteria vector vaccines [20,21].

Over the past three decades, significant advancements have been made in the research of ETEC vaccines, transitioning from conventional physical and chemical methods to cutting-edge genetic engineering techniques. Regrettably, thus far, no vaccine has demonstrated universal efficacy across all geographical regions. The majority of vaccine studies have encountered challenges attributed to suboptimal immunogenicity or limited broad-spectrum protection [22]. The current primary approach for preparing experimental and commercial ETEC vaccines involves the utilization of whole-bacteria-inactivated vaccines. However, this method is subject to limitations due to the extensive range of ETEC serotypes and regional disparities in distribution, resulting in restricted geographical applicability and inadequate broad-spectrum protection [23]. The inactivated vaccine also presents several limitations, including a low concentration of active ingredients, a high immune dosage, lack of efficacy, elevated endotoxin levels, and severe side effects, all of which have resulted in unsatisfactory clinical outcomes. On the other hand, the subunit vaccine has consistently attracted attention due to its advantages such as broad-spectrum potency, high immunogenicity, and minimal animal stress response. However, currently, there are challenges in strain expression level for preparing the bacterial antigen protein itself along with obtaining sufficient soluble protein and facing high purification costs. These obstacles have impeded its immune effectiveness and hindered its application in clinical practice thus far [24,25,26]. Hence, there exists an urgent necessity for an efficacious vaccine solution to prevent and control ETEC.

In this study, we present an oral vaccine utilizing a LAB vector for targeted delivering immunogens against F4+ ETEC. The administration of the vaccine through oral immunization obviates the stress reaction associated with injection, while concurrently promoting an efficient immune response and conferring protection against F4+ ETEC infection in animals.

## 2. Materials and Methods

### 2.1. Bacterial Culture

The *E. coli* C83539 strains were acquired from the China Center of Veterinary Culture Collection (CVCC) in Beijing, China. These strains were routinely cultivated aerobically at 37 °C in Luria–Bertani (LB) medium overnight. Meanwhile, LAB-dmLTA, LAB-LTB, LAB-faeG, and LAB empty vector strains were grown anaerobically in M17 medium (Hopebio, Qingdao, China) and agar containing 0.5% (wt/vol) lactose overnight at 30 °C.

### 2.2. Plasmid Construction and the Induction of Recombinant LAB

**Figure 1:**
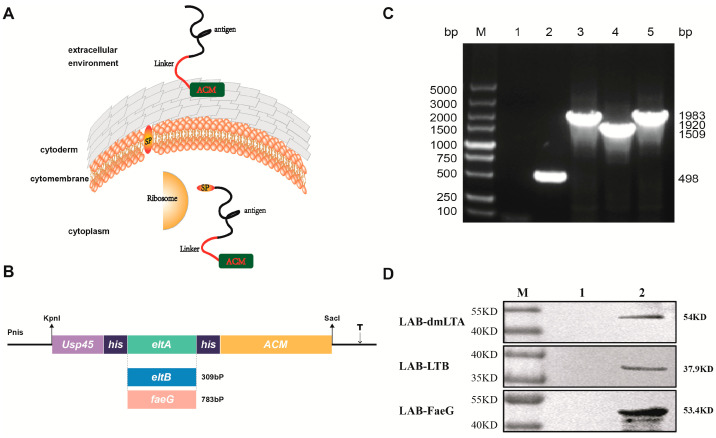
Construction of the recombinant lactic acid bacteria: (**A**) Schematic representation of surface-displayed antigens on lactic acid bacteria. (**B**) The construction scheme for the recombinant vector. (**C**) PCR identification of recombinant lactic acid bacteria. M: Marker; 1: negative control; 2: LAB; 3: LAB-dmltA; 4: LAB-ltB; 5: LAB-faeG. (**D**) WB identification of recombinant lactic acid bacteria. 1: NZ3900; 2: Three strains of recombinant LAB. Each displayed protein contains its ACM repeats at its C-terminus, and proteins are displayed on the surface of LAB.

As shown in Figure 1, The nisin-controlled gene expression (NICE) system strain NZ3900 of *Lactococcus lactis* subsp. lactis, harboring the regulatory genes *nis*R and *nis*K integrated into the antigen gene, was utilized as a vector for ETEC. Three surface display plasmids, namely pNZ8149-dmLTA, pNZ8149-LTB, and pNZ8149-faeG, were constructed. Electroporation was employed to transform *L. lactis* by applying a 2.2 kV electrical pulse with a resistance of 200 Ω and capacitance of 25 µF in a 0.2 cm cuvette using a Gene Pulser (Bio-Rad, Hercules, CA, USA). These LAB strains were designated as LAB-dmLTA, LAB-LTB, and LAB-faeG, respectively. As a negative control group, *L. lactis* was transformed with an empty shuttle vector to generate LAB strains without any recombinant plasmid insertion. To identify the three recombinant strains (LAB-dmLTA, LAB-LTB, and LAB-faeG), P213 MIX (GenScript, Nanjing, China) was used for PCR identification. The amplification reaction mixture contained 1 µL bacterial fluid sample, 1 µL forward primer (5′-ACGGCTC TGATTAAATTCTGAAGTT-3′), 1µL reverse primer (5′-GCTTTCATAATCTAACAGAC AACATCT-3′), 7 µL sterile water, and 10 µL of P213 MIX. The amplification procedure followed the instructions provided.

The single-cloned LAB-dmLTA, LAB-LTB, LAB-faeG, and control strain LAB empty vector were cultured in M17 liquid medium containing 0.5% lactose for 24 h. Subsequently, they were subcultured at a ratio of 1:25 for approximately 3–4 h until the OD_600_ value reached 0.4. At this point, the induction agent nisin was added to achieve a final concentration of 20 ng/mL, and the culture was continued for an additional 6 h.

### 2.3. Western Blot

The protein expression was evaluated through Western blot analysis. Cells were harvested by centrifugation at 12,000× *g* for 10 min, washed with precooled PBS, and then resuspended in a 1:10 dilution of PBS. The bacterial cells were disrupted using ultrasound for 15 cycles, with each cycle lasting 10 s. Subsequently, the disrupted bacterial solution was centrifuged at 12,000× *g* at 4 °C for 10 min and the supernatant was discarded. The resulting pellet was resuspended in PBS. These resuspended samples of the three recombinant strains and control bacteria were combined with a 5× protein loading buffer and subjected to heating at 100 °C for 10 min using a metal bath. SDS-PAGE protein electrophoresis was conducted on a 10% acrylamide gel. The gel was subsequently transferred onto polyvinylidene difluoride (PVDF) membranes (Merck Millipore, Billerica, MA, USA) using a Mini Trans-Blot Electrophoretic Transfer Cell (Bio-Rad, Hercules, CA, USA). Following blocking with a solution of 5% bovine serum albumin (BSA) for 2 h, the membranes were incubated overnight at 4 °C with an anti-His tag antibody (Biodragon, Shuzhou, China). After washing with PBST buffer solution, the membranes were incubated for 2 h with an HRP-conjugated goat antimouse IgG antibody (Biodragon, Shuzhou, China) diluted to a ratio of 1:5000. Reactivity was visualized using electrochemiluminescence reagents.

### 2.4. Oral Vaccine Preparation

Recombinant strains (LAB-dmLTA, LAB-LTB, LAB-faeG) were induced using the aforementioned strain induction protocol. Following induction, viable bacterial count was determined on a plate, resulting in an effective bacterial concentration of 3 × 10^9^ CFU/mL. Subsequently, the bacteria were mixed in a 1:1:1 ratio and centrifuged at 3000 rpm for 10 min. After centrifugation, the bacteria were washed once with PBS and resuspended in sterilized 15% skim milk. The suspension was further concentrated 10 times to obtain the composition of the LAB oral vaccine. The total concentration of LAB in this composition was 3 × 10^10^ CFU/mL, with each recombinant bacterium having a final concentration of 1 × 10^10^ CFU/mL. This composition was designated as ABG. Similarly, the empty vector strain vaccine consisting of LAB was prepared at the same concentration using identical methodology and named PNZ.

### 2.5. Mouse Immunoprotection Assay

**Figure 2:**
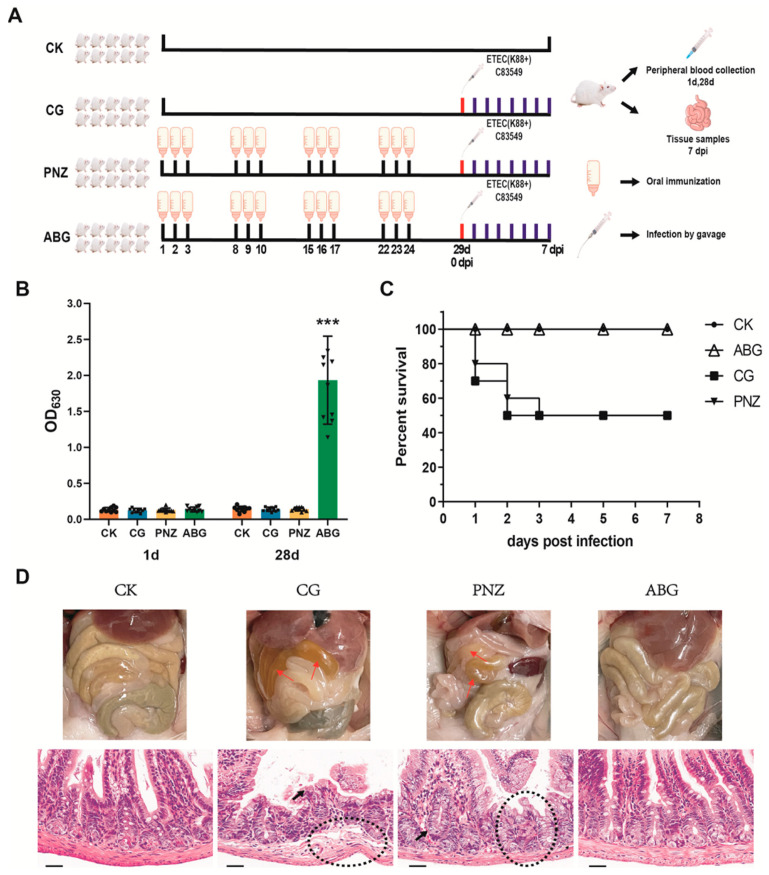
Immunological protective effect of a recombinant lactic acid bacteria live vector oral vaccine in mice: (**A**) Experimental protocol for the oral immunization assay in mice. (**B**) Serum IgG antibody levels before and after immunization in each group of mice (*** *p* ≤ 0.001). (**C**) Survival rate of mice in each group within 7 days after infection. (**D**) Anatomical intestinal lesions and HE staining results (40×) of small intestinal tissue pathological sections in each group of mice (The red arrows indicate the lesion site; Black arrows indicate inflammatory cells; The circles indicate the thinning of the bowel wall).

As shown in Figure 2, the BALB/c mice used in this study were obtained from the Laboratory Animal Services Center at Huazhong Agricultural University. Female BALB/c mice, aged 3–4 weeks, were randomly divided into four groups: a blank control group (CK, *n* = 10), an infected control group (CG, *n* = 10), a LAB empty vector strain vaccine group (PNZ, *n* = 10), and a LAB oral vaccine group (ABG, *n* = 10). The ABG group received the ABG vaccine orally for three consecutive days each week at a dose of 0.5 mL per mouse per day. They were then vaccinated for four weeks with a four-day interval. The PNZ and ABG groups followed the same immunization schedule but differed only in the use of the PNZ vector vaccine. On day 1 and day 28 of the experiment, peripheral blood samples were collected from all four groups of mice to determine serum IgG antibody levels using indirect ELISA. On day 29, the CG, PNZ, and ABG groups were infected by gavage with the F4+ ETEC standard strain C83549 at a dose of 5 × 10^8^ CFU per mouse; this time point was recorded as 0 days post infection (dpi). Mice were monitored for clinical signs and survival for seven consecutive days after challenge. All surviving mice were euthanized on 7 dpi and underwent autopsy; small intestines from all mice were collected for pathological sectioning and hematoxylin and eosin (H&E) staining.

### 2.6. Immunoprotection Assay for Piglets without Maternal Antibodies

**Figure 3:**
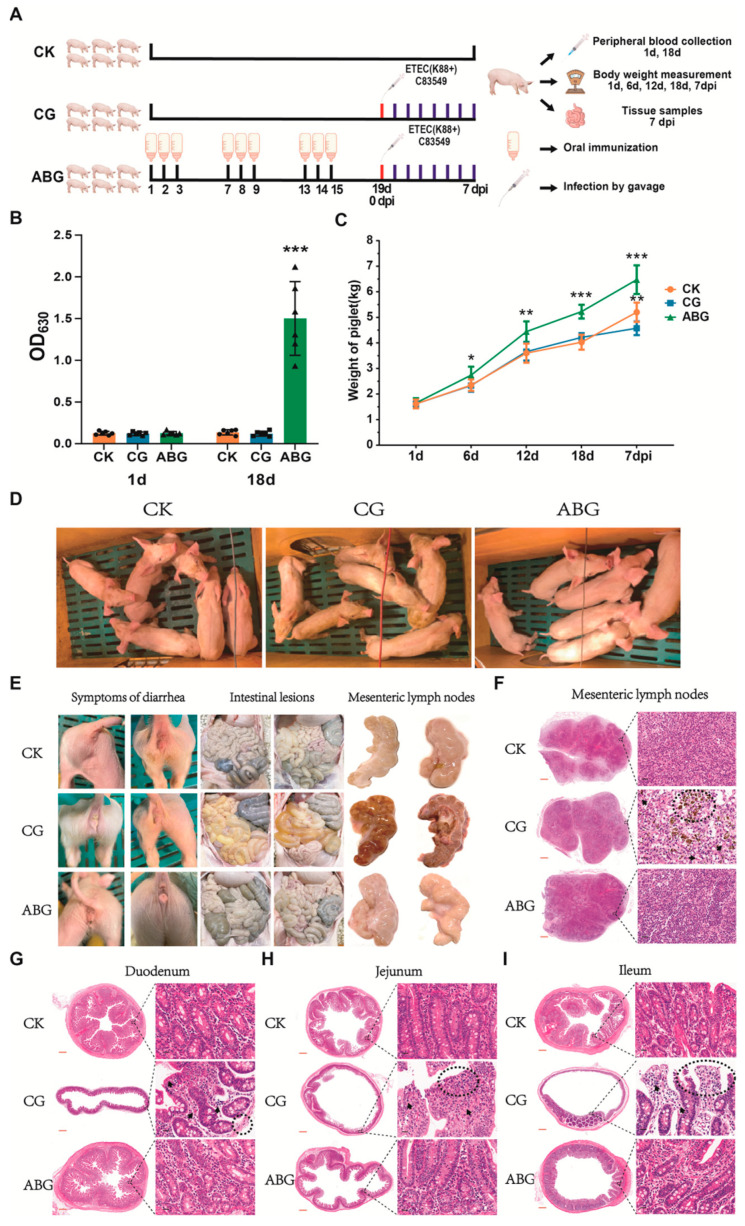
Immune protective effect of recombinant LAB live vector oral vaccine in piglets lacking maternal antibodies: (**A**) Experimental protocol for the animal experiment conducted on piglets without maternal antibodies. (**B**) Serum IgG antibody levels of piglets in each group before and after immunization (*** *p* ≤ 0.001). (**C**) The body weight of piglets throughout the experiment in each group (* *p* ≤ 0.05, ** *p* ≤ 0.01, *** *p* ≤ 0.001). (**D**) Clinical symptoms observed in piglets after infection for each group. (**E**) Diarrhea, intestinal anatomical lesions, and mesenteric lymph node lesions in piglets. (**F**) HE staining the pathological sections of mesenteric lymph node (red line: 1×, black line: 40×, the black arrows indicate inflammatory cells, the same as follows; The circle indicate hemosiderin deposition). (**G**) HE staining the pathological sections of duodenal (The circle indicate the thinning of the bowel wall). (**H**) HE staining the pathological sections of jejunal (The circle indicate accumulation of inflammatory cells). (**I**) HE staining the pathological sections of ileal (The circle indicate disrupted intestinal villi).

As shown in Figure 3, a total of 18 piglets aged 8 days were randomly allocated into three groups: the blank control group (CK, *n* = 6), the infected control group (CG, *n* = 6), and the LAB oral vaccine group (ABG, *n* = 6). The piglets in the ABG group received oral immunization with the ABG vaccine for three consecutive days in each round at a dose of 3 mL per piglet per day. This was followed by three rounds of immunization spaced three days apart. Peripheral blood samples were collected from all three groups of piglets on day 1 and day 18 of the experiment to determine serum IgG antibody levels using indirect ELISA. On day 19, both the CG and ABG groups were infected via gavage with the F4+ ETEC standard strain C83549 at a dose of 5 × 10^10^ CFU per piglet. This time point was recorded as 0 dpi. Clinical signs and diarrhea in the piglets were monitored for seven consecutive days after infection. The weights of all piglets were measured and recorded on days 1, 6, 12, 18, and 7 dpi during the experiment. At 7 dpi, all piglets were euthanized and necropsied. The duodenum, jejunum, ileum, and mesenteric lymph nodes from all piglets were collected.

### 2.7. Immunoprotection Assessment for Sows in Late Stage of Pregnancy

**Figure 4:**
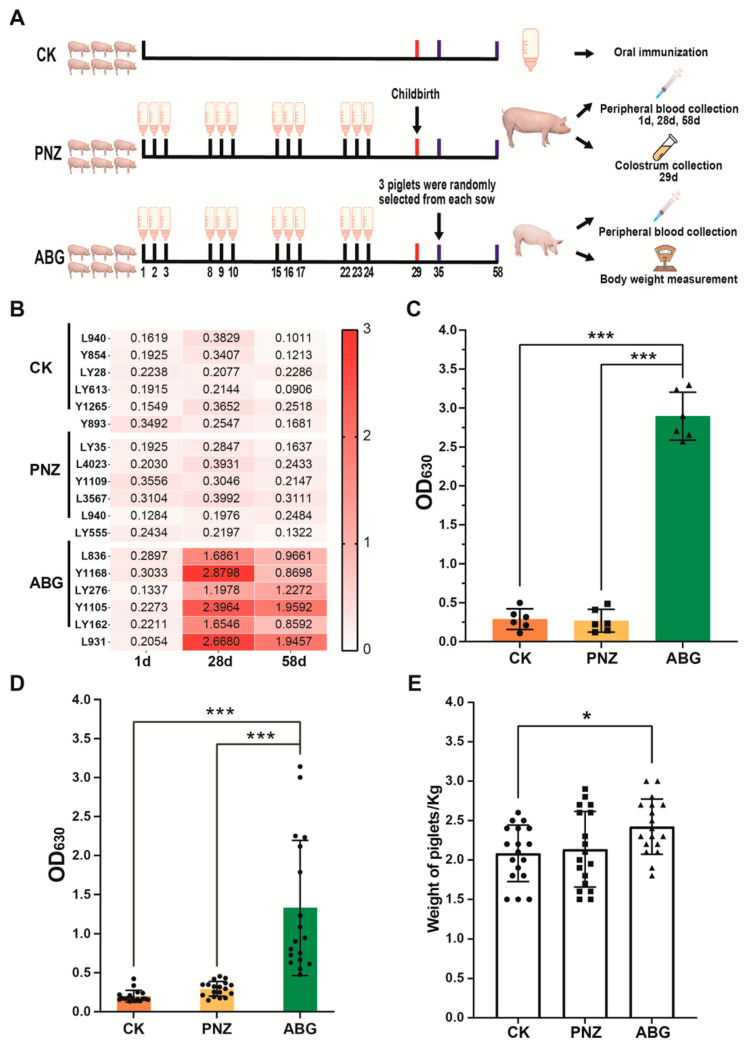
Immunogenicity of recombinant LAB live vector oral vaccine in pregnant sows: (**A**) Experimental protocol for the late-gestation sow trials. (**B**) Serum IgG antibody levels of sows in each group before and after immunization. (**C**) Colostrum IgG antibody levels in each group of sows after immunization (*** *p* ≤ 0.001, the same as follows). (**D**) Serum IgG antibody levels in piglets born to sows from each experimental gro. (**E**) Weight measurements of piglets born to sows from each experimental group (* *p* ≤ 0.05).

As shown in Figure 4, a total of 18 pregnant sows at day 86 of gestation were randomly allocated into 3 groups: the blank control group (CK, *n* = 6), the LAB empty vector strain vaccine group (PNZ, *n* = 6), and the LAB oral vaccine group (ABG, *n* = 6). The ABG group received oral immunization with ABG vaccine for three consecutive days per week at a dosage of 5 mL per sow per day, followed by a 4-day interval and immunization for 4 weeks. The PNZ and ABG groups followed the same vaccination schedule, except that the PNZ group used the PNZ vector vaccine. Fecal samples were collected from each pregnant sow in every group before and after immunization. Preimmunization samples were categorized as QCK, QPNZ, and QABG, while postimmunization samples were classified as HCK, HPNZ, and HABG. Peripheral blood samples were obtained from all three groups on days 1, 28, and 58 of experimentation to measure serum IgG antibody levels using indirect ELISA. All sows gave birth on day 29 of experimentation; colostrum was collected from each sow where whey was isolated to determine IgG antibody levels through indirect ELISA. On day 35 of experimentation, three piglets were randomly selected from each sow where they underwent weighing procedures, while peripheral blood samples were also taken to measure serum IgG antibody levels via indirect ELISA.

### 2.8. Immunoprotection Assay for Lactating Piglets with Maternal Antibodies

As shown in Figure 7, the 8-day-old piglets born to sows in Figure 4 were carefully selected and fed with artificial milk. Twelve piglets born to sows in the CK group were randomly divided into two groups: the blank control group (CK, *n* = 6) and the infected control group (CG, *n* = 6). Additionally, six piglets born to sows in the PNZ group were randomly chosen as the LAB blank vector strain vaccine group (PNZ, *n* = 6), while another six piglets born to sows in the ABG group were randomly assigned to the LAB oral vaccine group (ABG, *n* = 6). With the exception of the CK group, all three remaining groups of piglets underwent infection through gavage administration of F4+ ETEC standard strain C83549 at a dose of 1 × 10^10^ CFU per piglet, recorded as 0 dpi. Clinical signs and mortality of the piglets were monitored for 7 consecutive days after challenge. At 7 dpi, euthanasia was performed on all pigs followed by necropsy procedures. The duodenum, jejunum, ileum, and mesenteric lymph nodes from each individual piglet were collected.

### 2.9. Booster Immunization of Piglets with Maternal Antibodies

As shown in Figure 8, for the booster immunization of piglets, the pigs in CK, CG, and PNZ groups were selected in the same way as described above. Additionally, twelve piglets born to sows in the ABG group were randomly selected to form the booster immunization group (BIM, *n* = 6) and the ABG control group (*n* = 6). The BIM group received 3 mL per piglet per day of the ABG vaccine for 3 consecutive days during each round of immunization. Subsequently, they underwent 3 rounds of immunization with a 3-day interval. The PNZ group followed an identical immunization schedule but received the live-vector PNZ-inactivated vaccine instead.

The piglets in the ABG group continued receiving their mother’s milk until weaning and were treated at equivalent ages as other experimental groups. On day 1 and day 18 of the experiment, peripheral blood samples were collected from piglets in all five groups to determine serum IgG antibody levels using indirect ELISA. On day 19, gavage infection was performed on CG, PNZ, ABG, and BIM groups with F4+ ETEC standard strain C83549 at a dose of 5 × 10^10^ CFU per piglet; this time point was recorded as 0 dpi. Clinical signs and diarrhea episodes were monitored continuously for 7 days after infection. At 7 dpi, all piglets were euthanized and necropsied while collecting duodenum, jejunum, ileum, and mesenteric lymph nodes.

### 2.10. Enzyme-Linked Immunosorbent Assay (ELISA)

For the detection of IgG antibodies by indirect ELISA, flat-bottomed polystyrene plates were coated with dmLTA-his protein (100 ng/well in 0.1 M NaHCO_3_, pH 9.6) and incubated at 4 °C for 12 h, the dmLTA-his protein was purified by expression in *E. coli*. Subsequently, the plates were washed with phosphate-buffered saline (PBS) containing 0.05% Tween–20 (PBST). Then, each well was blocked by adding 250 μL of PBS containing 5% skim milk and incubating the plates at 37 °C for 2 h. After another round of washing with PBST, a sample (serum or whey), diluted to a ratio of 1:100 in PBS, was added to each well and incubated at 37 °C for 1 h. The plates were then washed again with PBST and further incubated at 37 °C for 1 h. Following this step, the plates were washed once more with PBST before being incubated with horseradish peroxidase (HRP)-conjugated goat antimouse IgG (Biodragon, Shuzhou, China) or goat antipig IgG (Biodragon, Shuzhou, China) (diluted to a ratio of 1:5000) at 37 °C for a duration of 45 min. After washing again with PBST, chromogenic solution was added to each well followed by the addition of stop solution after 15 min had elapsed. Finally, within 15 min from that point onwards, the absorbance value was measured at OD_630_ using a microplate reader.

### 2.11. Histopathological Examination

The tissues were collected, fixed in a 4% formaldehyde solution, and subsequently embedded in paraffin. Individual sections measuring 4 μm were then mounted on adhesive glass slides, dewaxed using xylene, and rehydrated with descending graded ethanol concentrations for H&E staining.

### 2.12. DNA Extraction and Microbiota Analysis

The HiPure Stool DNA Kits (model D3141, Guangzhou Magen Biotechnology Co., Ltd., Guangzhou, China) were utilized for fecal DNA extraction following the manufacturer’s protocol. Amplification of the 16S rDNA V3-V4 region was performed using the forward primer 341F (5′-CCTACGGGNGGCWGCAG-3′) and the reverse primer 806R (5′-GGACTACHV GGGTATCTAAT-3′). NanoDrop microvolume spectrophotometer (model NanoDrop 2000, Thermo Fisher Scientific, Waltham, MA, USA) was employed to assess DNA quality. The second round of amplification products was purified using AMPure XP Beads, quantified with the ABI StepOnePlus Real-Time PCR System (Thermo Fisher Scientific, Waltham, MA, USA), and sequenced in PE250 mode by pooling on the Novaseq 6000.

### 2.13. Data Analysis

Data were presented as mean ± standard error of the mean from at least three replicates. *t*-tests and one-way ANOVA analysis were conducted using GraphPad Prism 8.0 (GraphPad Software Inc., Bostoon, MA, USA). Statistical significance was considered at *p* ≤ 0.05, * *p* ≤ 0.05, ** *p* ≤ 0.01, *** *p* ≤ 0.001.

### 2.14. Ethics Statement

The animal procedures were conducted in accordance with the guidelines of the China Regulations for the Administration of Affairs Concerning Experimental Animals (1988) and Regulations for the Administration of Affairs Concerning Experimental Animals in Hubei (2005), following the protocol approved by the Animal Ethics Committee (No. 20230819). The experiments were carried out under strict routine management and specific pathogen-free conditions.

## 3. Results

### 3.1. Construction of Three Recombinant Lactic Acid Bacteria (LAB) Strains

The antigen was firmly attached to the surface of the LAB cell wall by non-covalent linkage, ensuring a stable display of foreign proteins (Figure 1A,B). The nisin-regulated expression (NICE) system strain NZ3900 of *Lactococcus lactis* subsp. lactis, which harbors the regulatory genes *nis*R and *nis*K integrated into the antigen gene, was employed as the vector for ETEC. Three surface display plasmids, namely pNZ8149-dmLTA, pNZ8149-LTB, and pNZ8149-faeG, were constructed. The gene sequence encoding the LTA (GenBank accession no. MF990203.1) subunit of ETEC heat-labile enterotoxin available in GenBank was analyzed. To mitigate toxicity associated with toxin protein expression, a mutation introducing lysine at position 63 (replacing serine) [27,28] and arginine at position 72 (replacing alanine) [29] in the LTA sequence resulted in an attenuated gene sequence named dmLTA. Additionally, gene sequences for the heat-labile enterotoxin subunit LTB (GenBank accession no. MF990203.1) and F4 (K88) pilin subunit faeG (GenBank accession no. DQ307495.1) were obtained from GenBank. To optimize protein presentation efficiency, the signal peptides in the three sequences were discarded, and the gene sequences were optimized according to the codon preference of *L. lactis* to obtain the antigen gene fragments for surface display. The secreted transmembrane peptide gene Usp45 [30] (GenBank accession no. EU382094.1) derived from lactic acid bacterial protein No. 45 served as a signal peptide for secretion of foreign proteins and was inserted at the 5′ end of each target gene fragment. Furthermore, a polypeptide fragment ACM (GenBank accession no. U17696.1) encoded by three repeated *lysM* [31] gene sequences derived from lactic acid bacteria was inserted at the 3′ end of each respective gene fragment. PCR identification results confirm the successful construction of recombinant plasmids (Figure 1C). All three recombinant LAB strains (LAB-dmLTA, LAB-LTB, and LAB-faeG) were successfully generated with sizes measuring 1983 bp, 1509 bp, and 1920 bp, respectively. Western blotting was performed to confirm the correct expression of foreign proteins in these recombinant strains; the bands corresponding to sizes of 54 KD (LAB-dmLTA), 37.9 KD (LAB-LTB), and 53.4 KD (LAB-faeG) were detected (Figure 1D).

### 3.2. Oral Immunization with a Recombinant LAB Live Vector Vaccine Protected Mice from F4+ ETEC Infection

The animal experimental scheme is depicted in Figure 2A. The results of serum IgG antibody detection demonstrate that prior to immunization (1 d), all mice exhibited no detectable antibodies. Following oral immunization (28 d), the ABG group displayed significantly elevated levels of antibodies compared with the CK, CG, and PNZ groups (*p* ≤ 0.001), with a notable enhancement in average levels (Figure 2B). Survival curve analysis revealed that within 7 days after challenge, 50% of mice in the CG and PNZ groups succumbed, whereas those in the ABG and CK groups survived without any discernible symptoms (Figure 2C). Anatomical examination and HE staining of histopathological sections unveiled intestinal wall thinning in both the CG and PNZ groups, accompanied by yellow fluid accumulation within the small intestine. Additionally, minor bleeding was observed within the small intestine of the CG group. Consistent with these anatomical findings, histopathologic sections stained with HE exhibited evident fragmentation and atrophy of intestinal villi, infiltration of intraepithelial lymphocytes, reduction in goblet cells and Paneth cells, and loss of plasma cells within the lamina propria, as well as abnormalities in muscle layer structure among mice from both the CG and PNZ groups (Figure 2D). In contrast, no lesions were detected within the intestines from mice belonging to either the ABG or CK group.

### 3.3. Recombinant LAB Live Vector Oral Vaccine Provides Protection against F4+ ETEC Infection in Weaned Piglets Lacking Maternal Antibodies

The experimental design is illustrated in Figure 3A. At the onset of the experiment (1d), serum IgG antibodies were initially assessed in piglets, and all piglets tested negative for antibodies, confirming the absence of maternal antibodies in piglets. Following oral immunization (18 d), the ABG group exhibited a significant elevation in piglet antibodies compared with the CK and CG groups (*p* ≤ 0.001) (Figure 3B). The weight gain of the piglets throughout the duration of the experiment is illustrated in Figure 3C. Following the initial round of immunization (6 days), there was a significant increase €n weight observed among piglets in the ABG group compared with both the CK and CG groups (*p* ≤ 0.05). Subsequent to the second round of immunization (12 days), piglets in the ABG group continued to exhibit a substantial increase in body weight, surpassing that of both the CK and CG groups (*p* ≤ 0.01). By the conclusion of three rounds of immunization (18 days), there was an even greater disparity in body weight. In comparison with the CK group, piglets in the ABG group experienced a 29.7% increase in body weight (*p* ≤ 0.001), while compared with those in the CG group, their body weight increased by 24.8% (*p* ≤ 0.001). The results at 7 dpi indicate that there was significant stagnation and a smaller growth rate observed among piglets within the CG group when compared with those within the CK group (*p* ≤ 0.01). However, following infection, piglets within the ABG group exhibited steady and continuous growth rates that were significantly higher than those observed within both CK and CG groups. The clinical symptoms observed in piglets from each group after infection are illustrated in Figure 3D. Piglets in the CG group exhibited signs of emaciation and rickets, whereas those in the ABG group showed no symptoms and even demonstrated superior growth compared with the CK group. The results of diarrhea and anatomy in piglets from each group after infection are depicted in Figure 3E. It is evident that significant diarrhea was observed among piglets in the CG group, while both the ABG and CK groups remained symptom-free. An analysis of intestinal lesions revealed notable manifestations such as thinning of the intestinal wall, transparency, and gas bulges among piglets in the CG group. Specifically, yellow liquid and foamy contents were found to fill segments of their small intestine. In contrast, no lesions were detected within the intestines of either the ABG or CK groups. The results obtained from examining mesenteric lymph nodes indicate obvious bleeding, swelling, and other lesions within the CG group; however, normal findings were observed for both the ABG and CK groups. The results of HE staining of pathological sections of mesenteric lymph nodes are presented in Figure 3F. The yellow signal observed in the CG group image indicates hemosiderin deposition resulting from red blood cell phagocytosis. Compared with the CK group, the CG group exhibited a decrease in mature lymphocytes, while the ABG group showed an increase in mature lymphocytes within the tissue. Pathological sections of piglet duodenum, jejunum, and ileum stained with HE are shown in Figure 3G–I. In the CG group, notable findings included thinning of the intestinal wall muscular layer, intestinal mucosal atrophy and fragmentation, hemorrhage, intestinal villus necrosis, infiltration of intraepithelial lymphocytes, and loss of plasma cells in the lamina propria, among other lesions. Conversely, both ABG and CK groups displayed normal results.

### 3.4. Immunization of Sows with the Recombinant LAB Live Vector Oral Vaccine during Late Pregnancy Effectively Enhances the Transfer of Maternal Antibodies to Piglets

According to the experimental design depicted in Figure 4A, sows were immunized at 86 days of gestation. The results indicate an absence of ETEC antibodies in the sows prior to immunization. Following four rounds of immunization (28 days), a significant increase in antibodies was observed in the ABG group, while no antibody production was detected in the PNZ group. After 30 days of immunization (58 days), antibody levels were reassessed for each group. The ABG group still exhibited elevated antibody levels, albeit slightly lower than those observed at day 28 (Figure 4B). On day 29 of the experiment, all sows gave birth within their respective groups. Colostrum samples were collected, and whey was isolated to measure antibody levels. Results demonstrate that colostrum from sows in the ABG group had significantly higher antibody content compared with both CK and PNZ groups (*p* ≤ 0.001) (Figure 4C). On day 35 of the experiment, three piglets born to each sow were randomly selected and grouped based on their respective sow groups. Blood samples were collected from all piglets for antibody detection and weight measurements. The results of IgG antibody detection are presented in Figure 4D. Piglets born to sows in the ABG group exhibited significantly higher levels of antibodies compared with those in the CK and PNZ groups (*p* ≤ 0.001), although there was greater variability within the ABG group. The body weight results are depicted in Figure 4E. Piglets born to sows in the ABG group demonstrated significantly higher weights than those in the CK and PNZ groups (*p* ≤ 0.05).

### 3.5. Immunization of the Recombinant LAB Live Vector Oral Vaccine Significantly Enhanced the Abundance of Lactobacillus in the Intestinal Tract of Sows during Late Pregnancy

**Figure 5:**
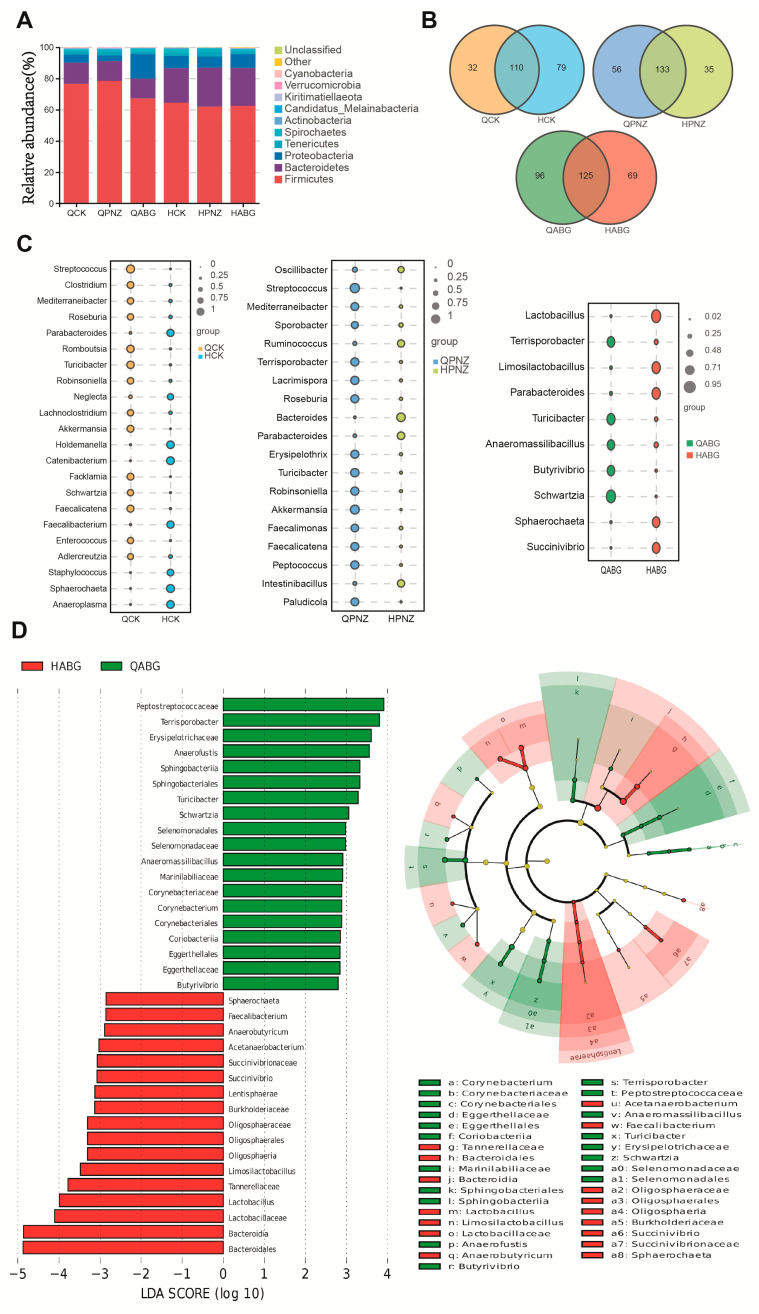

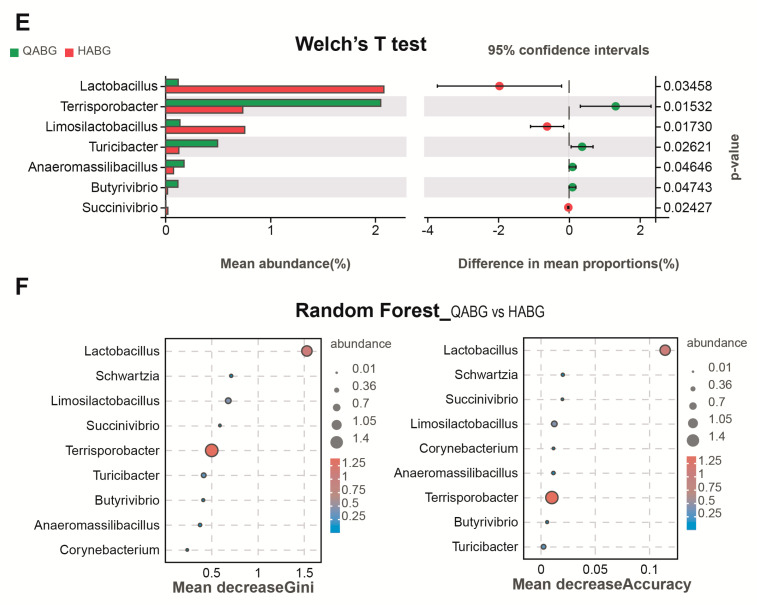
Analysis of intestinal flora community structure in pregnant sows before and after oral immunization: (**A**) Stacked plot of sow fecal microbiota at the phylum taxonomic level. (**B**) The Venn diagram illustrates the similarities and differences in the genus-level composition of fecal microbial communities before and after immunization within each group. (**C**) Indicator analysis of the differences in fecal microbial community at the genus level before and after immunization within each group. Only species exhibiting significant differences (*p* ≤ 0.05) are presented, with bubble size indicating their corresponding indicator value. (**D**) The fecal microbial community at the genus level in the ABG group was analyzed using LEFSE to identify species differences before and after immunization. Statistical analysis was conducted using a two-sided Wilcoxon rank sum test. The final differences were ranked based on Linear Discriminant Analysis (LDA) results, with a retention threshold of LDA score ≥ 2 by default, resulting in the left figure. Subsequently, these differences were mapped onto a classification tree with known hierarchical structure to generate an evolutionary branch map, as shown in the right figure. (**E**) Welch’s *t*-test results of fecal microbial community at the genus level before and after immunization in the ABG group were analyzed. The left half of the figure represents different species, while the mean abundance of each species is shown on the x-axis. On the right half, the x-axis indicates differences in abundance between groups, with dot color indicating higher abundance in a specific group. The error bar around each dot represents the 95% confidence interval fluctuation range for that difference. Additionally, significance (*p*-value) of intergroup differences for corresponding species is indicated on the y-axis. (**F**) The genus-level fecal microbial communities in the ABG group were analyzed using random forest to investigate species differences before and after immunization. The results, presented as Gini index and Mean Decrease Accuracy, are depicted by bubble size and color representing species abundance, while the abscissa indicates the corresponding size of the index.

In the pregnant sow experiments, fecal samples were collected from each group of sows before (1 d) and after (28 d) immunization. Then, 16s rDNA was extracted from these samples for sequencing analysis. The results of the sow fecal microbiota at the phylum classification level are presented in Figure 5A. It was observed that the microbial composition of all groups was identical, indicating that the samples met the requirements for further analysis. To further assess differences in gut microbiota at the genus level, we analyzed microbiota before and after immunization in each group, as shown in Figure 5B. The gut microbiota of each group exhibited variations on day 1 and day 28 of the experiment, with differences noted between different groups. The indicator analysis in Figure 5C illustrates the variations in bacterial groups at the genus level among the three groups before and after immunization. Bubble size represents bacterial species content, with only significantly different species displayed. Results indicate a significant increase in *Lactobacillus* exclusively within the ABG group post immunization, while no presence of *Lactobacillus* was observed in the CK and PNZ groups’ differential flora. Furthermore, LEFSE analysis was conducted on the differential bacterial groups at the genus level pre and post immunization, specifically within the ABG group. The results reveal notable differences in bacterial species before and after immunization, particularly showcasing a substantial rise in *Lactobacillus* proportion ranking third following immunization. These results align with those obtained from indicator analysis (Figure 5D). The results of Welch’s *t*-test confirm the previous findings regarding the differential bacterial flora levels in the ABG group before and after immunization. The average abundance data demonstrated a significant increase in *Lactobacillus* abundance after immunization compared with before, with the most pronounced difference observed among all strains (Figure 5E). A random forest analysis was conducted at the genus level on the differential bacterial flora of the ABG group pre and post immunization. The analysis employed both the Gini index and the average precision reduction index. The Gini index reflects the purity of the decision tree nodes, with a higher value indicating better discrimination between groups. The average accuracy reduction, measured by the mean square error, indicates the impact of randomly assigning values to variables on the accuracy of the prediction results. A higher value suggests a stronger indicator effect for distinguishing between groups. In Figure 5F, bubble size and color represent species abundance, while bubble position reflects index magnitude. Although there were differences in results obtained from these two indices, both identified *Lactobacillus* as having the highest index and greater abundance within its genus (Figure 5F).

### 3.6. The Richness and Uniformity of the Intestinal Microbiota in Late-Pregnant Sows Were Improved after Four Rounds of Immunization with a Recombinant Live LAB Vector Oral Vaccine

**Figure 6:**
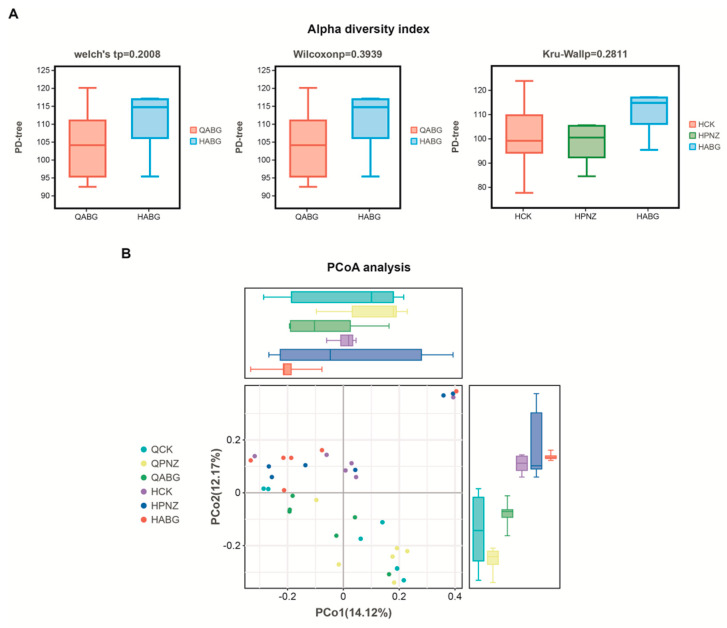
Diversity analysis of intestinal flora community in three groups of pregnant sows before and after oral immunization: (**A**) The α-diversity of fecal microbiota was analyzed before and after immunization in each group. The PD-whole tree index, which evaluates lineage diversity based on the phylogenetic features of the OTU sequence evolutionary tree, was used. The y-axis represents the alpha diversity index, with the graph displaying the maximum (top line), minimum (bottom line), median (middle box), upper quartile (top box), lower quartile (bottom box), and outliers outside these ranges. Welch’s *t*-test and Wilcoxon rank sum test were performed to assess differences in α diversity of fecal microbiota before and after immunization in the ABG group, while Kruskal–Wallis’s rank sum test was used to evaluate differences in α diversity of fecal microbiota after immunization among the three groups. (**B**) The results of principal coordinate analysis (PCoA) are presented. The PCo1 coordinate represents the first principal component, with the percentage in parentheses indicating its contribution to sample variance. Similarly, the PCo2 coordinates represent the second principal component, and the percentages in parentheses indicate their contribution to sample differences. Each sample is represented by colored points in the figure. In these analysis results, closer distances on the PCoA figure reflect greater similarity between samples.

The assessment of alpha diversity commonly relies on two crucial parameters, species richness (the number of species) and species evenness (their distribution), which are used to determine the diversity within a given habitat or ecosystem. A higher number of species and a more balanced distribution indicate a superior sample quality and a greater degree of diversity. To evaluate the extent of diversity, the PD tree index is employed, which assesses lineage diversity based on the phylogenetic characteristics derived from OTU sequence evolutionary trees. In this study, we utilized Welch’s *t*-test and Wilcoxon rank sum test to analyze changes in species diversity within the ABG group before and after immunization. The results reveal an increase in diversity following immunization for the ABG group. Additionally, a Kruskal–Wallis rank sum test was conducted to compare diversities among three groups (HCK, HPNZ, and HABG) post vaccination. Notably, the HABG group exhibited the highest level of diversity, suggesting that supplementation with live vector recombinant LAB oral vaccine enhanced gut flora diversity in sows (Figure 6A).

Beta diversity refers to the variation in species diversity across ecosystems, specifically when comparing bacterial community structures between samples or groups. The β-diversity index takes into account both the presence or absence of species and their abundance. In our analysis, we utilize the Bray_Curtis index to quantify this diversity. However, interpreting the results of this distance index can be challenging due to its abstract and intricate nature. To address this challenge, we employ principal coordinate analysis (PCoA) to visually represent the dissimilarities in bacterial community structure between samples. PCoA is a dimensionality reduction technique that effectively highlights key differences within complex datasets. Therefore, our results primarily focus on reflecting dissimilarity distances between samples. In the PCoA plot, closer sample points indicate higher similarity levels among them. Our analysis reveals that among the six sample groups examined, postimmunization ABG exhibits minimal disparities in PCo1 and PCo2 coordinates, indicating a high degree of similarity between these groups (Figure 6B).

### 3.7. Maternal Antibodies from Orally Immunized Sows Protected Suckling Piglets (8 d of Age) from F4+ ETEC Infection

**Figure 7:**
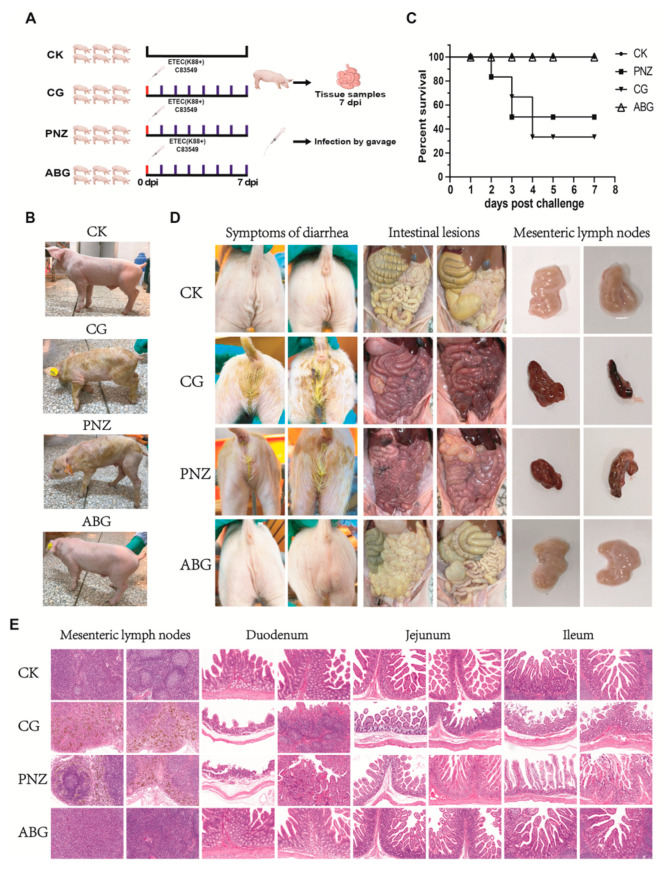
Results of ETEC infection in piglets with maternal antibodies during lactation: (**A**) Protocol for animal experiments during the suckling stage of piglets with maternal antibodies. (**B**) Clinical symptoms observed in each group of infected piglets. (**C**) Survival rates of piglets within 7 days after infection in each group. (**D**) Diarrhea, intestinal pathological changes, and mesenteric lymph node pathological changes in piglets. (**E**) Histopathological results from HE staining sections of mesenteric lymph nodes, duodenum, jejunum, and ileum in piglets (10×).

The infection assay was performed as shown in Figure 7A. Piglets were orally infected at 8 days of age and monitored for a period of 7 days post infection. The clinical manifestations exhibited by the piglets after infection are depicted in Figure 7B. Severe symptoms, including diarrhea, dehydration, weight loss, rickets, and tremors, were observed in piglets belonging to the CG and PNZ groups. Conversely, no abnormal symptoms were observed in piglets from the CK and ABG groups. The survival curve within the 7-day infection period is presented in Figure 7C. In the CG group, four piglets succumbed to the infection, resulting in a survival rate of 33.3%. Similarly, three piglets died in the PNZ group, leading to a survival rate of 50%. In contrast, all piglets from both ABG and CK groups survived with a perfect survival rate of 100%. The diarrheal symptoms and anatomical observations of the piglets in each group after infection are depicted in Figure 7D. Notably, piglets in the CG and PNZ groups exhibited pronounced yellow watery diarrhea, while no signs of diarrhea were observed in the ABG and CK groups. Intestinal anatomy analysis revealed that piglets in the CG and PNZ groups displayed symptoms such as hemorrhage, dehydration, intestinal wall thinning, and fluid accumulation. Conversely, the intestines of the ABG group and CK group appeared relatively normal. Mesenteric lymph node examination demonstrated significant hemorrhage and atrophy in the CG group, whereas both ABG and CK groups exhibited normal lymph nodes. The HE staining results of pathological sections of mesenteric lymph nodes, duodenum, jejunum, and ileum in piglets are presented in Figure 7E. In the mesenteric lymph nodes, both the CG and PNZ groups exhibited a significant presence of yellow signals indicating hemosiderin deposition resulting from red blood cell engulfment, confirming bleeding occurrence in these two groups of mesenteric lymph nodes. Compared with the CK group, the CG and PNZ groups showed a notable decrease in mature lymphocyte count. Conversely, the ABG group demonstrated a higher number of mature lymphocytes compared with the CK group. HE staining results for pathological sections of the duodenum, jejunum, and ileum reveal evident congestion, hemorrhage, thinning of intestinal wall muscular layer, and atrophy and fragmentation of intestinal mucosa, as well as intestinal villus necrosis in both the CG and PNZ groups. However, no abnormalities such as intraepithelial lymphocyte infiltration or loss of plasma cells were observed in either the ABG or CK groups.

### 3.8. Booster Immunization of the Live Oral Recombinant LAB Vector Vaccine in Piglets with Maternal Antibodies during Lactation Enhances Their Resistance against F4+ ETEC Infection at Weaning

**Figure 8:**
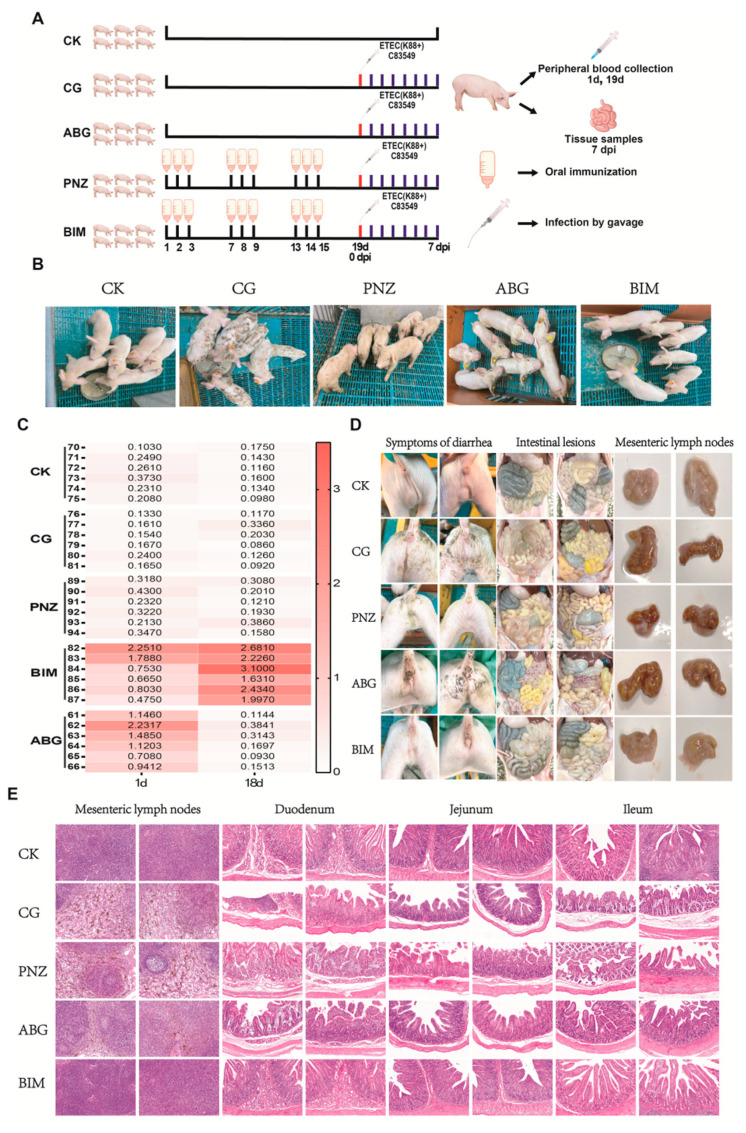
The immune protective effect of an oral vaccine booster using recombinant LAB in piglets with maternal antibodies: (**A**) Experimental protocol for the weaned piglets with/without booster immunization. (**B**) Clinical symptoms of piglets in each group after infection. (**C**) Serum IgG antibody levels measured before and after booster immunization in each group of piglets. (**D**) Diarrhea, intestinal lesions, and mesenteric lymph node lesions observed in each group of piglets. (**E**) HE staining the pathological sections of mesenteric lymph nodes, duodenum, jejunum, and ileum obtained from each group of piglets (10×).

The booster immunization assay in piglets with maternal antibodies is depicted in Figure 8A. The clinical symptoms of the infected piglets are illustrated in Figure 8B. Both the CG group and the PNZ group of piglets exhibited symptoms such as diarrhea, weight loss, and rickets, while the piglets in the CK group and BIM group remained asymptomatic. The results of the antibody detection are presented in Figure 8C. On the first day of the experiment, serum IgG antibody detection results indicate the presence of maternal antibodies in both the ABG group and BIM group piglets. On day 18 of the experiment, serum IgG antibody results show that the BIM group exhibited sustained elevation in serum antibody levels, whereas the ABG group’s initially high maternal antibodies had diminished by this time and were comparable to those observed in the CK, CG, and PNZ groups. The diarrheal symptoms and anatomical changes in piglets after infection are depicted in Figure 8D. Diarrheal symptoms were observed in the CG, PNZ, and ABG groups, while the BIM and CK groups remained unaffected. Intestinal anatomy analysis revealed significant thinning of the intestinal wall, the presence of yellow fluid content, bloating, bleeding, and other symptoms in the CG group, PNZ group, and ABG group. Conversely, the intestines of the BIM group and CK group appeared relatively normal. An evaluation of the mesenteric lymph nodes demonstrated noticeable bleeding in the CG group, PNZ group, and ABG group; however, normal results were obtained for the BIM group and CK group. The HE staining results of pathological sections from the mesenteric lymph node, duodenum, jejunum, and ileum in each group are presented in Figure 8E. In both the CG group and PNZ group, there was a significant accumulation of hemosiderin deposition observed in the mesenteric lymph node, along with a notable decrease in mature lymphocytes. The ABG group also exhibited these characteristics, albeit with slightly milder symptoms compared with the first two groups. Conversely, both the BIM group and CK group displayed relatively normal findings. Regarding the HE staining results of pathological sections from the duodenum, jejunum, and ileum, the thinning of the intestinal wall muscular layer, atrophy and fragmentation of the intestinal mucosa, and necrosis of intestinal villi were evident in both the CG group and PNZ group, along with infiltration of intraepithelial lymphocytes. The ABG group also demonstrated similar symptoms but to a lesser extent than those seen in the first two groups. In contrast, both the BIM and CK groups showed normal characteristics.

## 4. Discussion

ETEC is a well-documented etiological agent of diarrhea and mortality in various juvenile animals, including humans. Its high prevalence among piglets has resulted in substantial economic losses within the swine industry [27]. Among the various types of pili, F4+ ETEC is particularly concerning due to its potential to cause acute diarrhea and even mortality in neonatal, suckling, and weaned piglets. F4+ ETEC exhibits a higher prevalence rate and results in greater economic losses compared with other pilus variants [32]. Upon entering the animal’s body, the strain initially adheres to the small intestinal mucosa through pili. Subsequently, it secretes heat-labile (LT) and/or heat-stable enterotoxin (ST), resulting in diarrhea or severe disease [33]. FaeG, as the primary subunit of the F4 adhesin, has been observed to elicit robust pilus-specific mucosal and systemic immune responses. This suggests its potential as a promising immunogen candidate for developing vaccines against F4+ ETEC infection [27,34,35,36]. The A and B subunits of the LT have been evaluated explicitly by researchers, revealing their inherent immunogenicity and ability to confer immune protection. When administered in combination, their synergistic effect surpasses that of individual administration. Notably, forming the AB5 structure is not required for these subunits. Consequently, incorporating both subunit epitopes into an ETEC vaccine candidate is more likely to elicit neutralizing responses against LT toxin compared with using either subunit alone [37,38].

Since the 1990s, there has been a growing interest in studying LAB as a potential delivery system for various biomolecules [39,40]. LAB offers several advantages, including safety, cost-effectiveness, ease of use and genetic manipulation, scalability for industrial production, and the ability to elicit robust systemic and mucosal immune responses. Consequently, researchers investigating mucosal vaccines have taken notice [41]. Previous studies have demonstrated that administering recombinant LAB expressing antigens via oral or nasal routes can effectively induce mucosal immune responses and confer protection against diverse bacteria and viruses [39,42,43]. Therefore, this study employed *Lactococcus lactis* as the expression vector to individually display each of the three antigens of F4+ ETEC on its surface and developed the mixed recombinant LAB vaccines, with the aim of evaluating its potential as an oral vaccine candidate for the prevention of F4+ ETEC infection. Here, LTA, LTB, and FaeG were selected as the candidate immunogens for this study, among which the amino acids 63 and 72 of the LTA subunit were mutated to reduce its toxicity [27,28,29], while the LTB subunit remained unaltered. FaeG, the main adhesion subunit of the F4 (K88) pilus, was also chosen. A secreted transmembrane peptide gene *Usp45* [30,44] and the cell wall non-covalent linking motif *LysM* [31,45,46], both derived from LAB itself, were used to present these antigens on the surface of LAB. Codon optimization was employed to enhance expression levels. Using this protocol, three recombinant LAB strains successfully displayed these aforementioned antigens on their surfaces (Figure 1).

In the current work, recombinant LAB strains were combined to develop an oral vaccine, which was subsequently evaluated for its protective efficacy in mice and pigs. The experiments conducted on mice revealed that after four rounds of oral immunization, the mice exhibited elevated levels of serum IgG antibodies and complete protection against exposure to a 50% lethal dose of F4+ ETEC. The results obtained from HE staining on tissue sections from the surviving mice confirm these findings, providing further evidence supporting the notion that this oral vaccine can induce antibody production capable of effectively combating F4+ ETEC infection in mice, as previously observed in other studies [47,48].

Another study focused on the effects of oral vaccination on piglets lacking maternal antibodies. The experiment involved 8-day-old suckling piglets, specifically selected for the study. Prior to commencing the experiment, the serum IgG antibody levels of the piglets were assessed to ensure the absence of any maternal antibodies. Subsequently, they were separated from sow feeding and provided with artificial milk powder as their source of nutrition. The results reveal that after three rounds of immunization, significant weight gain was observed in the piglets along with elevated levels of serum IgG antibodies [49]. Furthermore, when exposed to a full dose of diarrhea, these immunized piglets demonstrated protection against it [50]. The body weight results following exposure indicate continued positive growth in the immunized piglets without being affected by infection. Autopsy findings further confirmed the effectiveness of oral vaccination in providing protective immunity to these young pigs. In conclusion, these findings strongly support that oral vaccination induces protective immunity in piglets lacking maternal antibodies.

More importantly, the most detrimental stage of ETEC infection in pigs is during the neonatal piglet phase, which primarily induces severe diarrhea and dehydration, often resulting in mortality among piglets under 7 days old. To protect piglets during this critical period, acquiring higher levels of maternal antibodies from the sow is imperative as they offer effective protection [51,52]. Therefore, we implemented oral immunization of sows one month prior to parturition. The results reveal that after four rounds of oral immunization, sows attained elevated serum IgG antibody levels. The antibody levels remained relatively high even at 30 days post immunization (the weaning stage for piglets). Similar outcomes were observed when assessing the antibody levels in colostrum obtained from immunized sows. Colostrum derived from immunized sows exhibited significantly higher antibody concentrations compared to the other two groups, with levels approximately twice as high as those found in serum samples. The serum antibody levels in piglets born from immunized sows also demonstrated significant improvement; however, variations were noticeable among individual piglets due to differences in colostrum intake. Additionally, this study evaluated the body weight of piglets born to sows within each group at 8 days old. The results indicate that offspring born to immunized sows displayed significantly greater body weights compared with those born to nonimmunized counterparts or control groups. This suggests that oral vaccines not only deliver antigens but also positively impact litter weight gain among piglets (Figure 4).

Moreover, the results of this study reveal a significant increase in the abundance of *Lactobacillus* in the intestinal microbiota of sows following immunization. Notably, the administration of oral vaccines concurrently led to an increase in the abundance of other beneficial bacteria, whereas a higher presence of harmful bacteria was observed in the intestines of preimmunized sows. This indicates that the utilization of oral vaccines induces alterations in the intestinal milieu of sows. Similarly, the analysis of bacterial alpha diversity demonstrated an enhancement in intestinal flora diversity within the oral vaccine group, potentially due to the utilization of carrier LAB strains employed in this vaccine formulation. The increased abundance of this particular group promotes improved intestinal health among animals. Furthermore, an analysis comparing beta diversity differences indicated that the composition of intestinal flora in pigs after oral vaccine immunization exhibited similarity and the highest uniformity among all groups. This confirms that the inclusion of live vector vaccines directly enhances intestinal health among pigs and consequently explains the higher weight observed for piglets born from vaccinated sows compared with the control group. However, *L. lactis* was not found in the differential flora, indicating that *L. lactis* could not colonize the gastrointestinal tract for a long time. Relevant studies have shown that *L. lactis* can only survive for 4 h in the human gastrointestinal tract (GIT) [53], and when dead in the gastrointestinal tract, it cannot spread in the environment, which makes it safer as a genetically modified organism (GMO) [45,49,54].

The present study conducted a challenge test on piglets born from immunized sows to investigate the efficacy of maternal antibodies in protecting against ETEC infection. The results demonstrate that piglets with maternal antibodies exhibited resistance to a half-lethal dose attack, as all the piglets in the immune group survived, compared with the 60% mortality rate in the control group. These findings indicate that oral vaccination of sows can confer newborn piglets with maternal antibodies, enabling them to resist ETEC infection. However, it was observed that maternal antibodies did not provide protection against ETEC infection during the weaning stage. Maternal antibody levels gradually declined throughout lactation and were completely absent by the time of weaning (Figure 8C) [51]. Based on these findings, an enhanced immunization program was implemented for piglets with existing maternal antibodies starting at 8 days of age. After three rounds of strengthened immunization, there was further improvement in serum IgG antibody levels during the weaning stage for these piglets (Figure 8C). The group receiving boosted immunization achieved complete protection against diarrhea, while both the unboosted maternal antibody group and the group without maternal antibodies experienced episodes of diarrhea (Figure 8D). This confirms the effectiveness of boosted immunization in safeguarding piglets during weaning. Overall, these experiments demonstrate that recombinant LAB live vector oral vaccine effectively protects piglets against F4+ ETEC infection by administering four rounds of oral immunization to pregnant sows one month prior to parturition and subsequently providing three additional booster rounds of oral immunization during the lactation period until weaning.

However, there are limitations to this study. The booster immunity test involved the separation of piglets from sows and their feeding with artificial milk powder, which may have excluded the potential influence of antibodies in breast milk on oral booster immunity [55]. Further experiments are required to confirm this potential impact. Additionally, this study did not evaluate cross-protection against other prevalent strains, such as the F18 fimbriae type that causes disease in piglets. Therefore, additional experimental verification is necessary for this aspect. Overall, the oral vaccine demonstrated effectiveness in immunizing sows and enabling piglets to acquire maternal antibodies, thereby protecting them from F4+ ETEC infection. After boosting immunization during lactation, it also provided strong protection against infection in piglets after weaning. Furthermore, even without maternal antibodies present, the oral immunization of piglets alone was found to protect them from diarrhea during the weaning stage. Henceforth, this recombinant LAB live vector oral vaccine exhibits great potential for protecting piglets throughout their susceptible stage to ETEC and holds promising prospects for future development.

## 5. Conclusions

In this study, three recombinant LAB were created by presenting the three antigens of F4+ ETEC on the surface of *Lactococcus lactis* and formulated into an oral vaccine in equal proportions. Animal experiments confirmed that this oral vaccine not only enhances the resistance of mice against F4+ ETEC infection but also assists piglets lacking maternal antibodies in combating PWD caused by F4+ ETEC. Immunizing sows with this oral vaccine in late pregnancy allows piglets to acquire maternal antibodies after birth, enhancing their defense against F4+ ETEC during lactation. However, these acquired antibodies are insufficient in effectively combating F4+ ETEC-induced diarrhea during weaning. Therefore, we utilized this oral vaccine as a booster for piglets containing maternal antibodies during lactation, which was similarly able to help piglets resist PWD caused by F4+ ETEC. These findings suggest that when administered through a well-designed oral immunization schedule, the recombinant LAB live vector oral vaccine described in this study can effectively safeguard piglets from F4+ ETEC infection during both lactation and weaning.

## Data Availability

All data are available in this manuscript. Additional information can be obtained from the corresponding authors upon reasonable request.
